# Role of STX1A in Parkinson’s disease: evidence, mechanistic hypotheses, and translational perspectives

**DOI:** 10.3389/fnagi.2026.1905708

**Published:** 2026-08-17

**Authors:** Rui Xu, Rui Li, Hongmei Li, Qingyun Li, Yanping Li, Gang Wu, Xiaolin Dong

**Affiliations:** Department of Neurology, The Affiliated Yan’An Hospital of Kunming Medical University, Kunming, Yunnan, China

**Keywords:** biomarker, calcium signaling, Parkinson’s disease, SNARE complex, synaptic dysfunction, syntaxin-1A

## Abstract

Parkinson’s disease (PD) is a progressive neurodegenerative disorder characterized by degeneration of substantia nigra dopaminergic neurons, α-synuclein pathology, and widespread synaptic dysfunction. Syntaxin-1A (STX1A), a presynaptic Qa-SNARE protein, is essential for synaptic vesicle fusion and also interacts with proteins involved in ion-channel regulation and neuronal excitability. Several animal, extracellular-vesicle, and peripheral-blood studies have reported reduced STX1A abundance in PD. However, the available evidence is predominantly cross-sectional and does not establish whether STX1A downregulation is a cause of PD pathology, a consequence of neuronal and synaptic loss, or a peripheral correlate of disease. This narrative review critically evaluates evidence linking STX1A to PD and distinguishes experimentally supported observations from mechanistic hypotheses. Potential relationships with calcium dysregulation, mitochondrial injury, ferroptosis, and neuroimmune signaling are discussed as testable models rather than established pathways. Direct evidence connecting STX1A to the gut–brain axis in PD is currently lacking and is therefore considered primarily as a future research direction. The biomarker and therapeutic potential of STX1A remains preliminary because diagnostic performance, disease specificity, longitudinal stability, and causal relevance have not been adequately validated. Future studies using cell-type-specific STX1A manipulation, rescue experiments, electrophysiology, and multicenter longitudinal cohorts are required to define the biological and clinical significance of STX1A in PD.

## 1 Introduction

Parkinson’s disease (PD) is the second most common neurodegenerative disorder and is clinically characterized by motor and non-motor manifestations. Its major pathological features include progressive loss of dopaminergic neurons in the substantia nigra pars compacta and abnormal accumulation of α-synuclein. Mitochondrial dysfunction, oxidative stress, impaired proteostasis, neuroinflammation, and synaptic failure are interconnected components of PD pathophysiology. Current treatments, including dopaminergic replacement and deep brain stimulation, improve symptoms but do not reliably halt neurodegeneration (Deliz et al., 2024; Armstrong and Okun, 2020; Costa et al., 2023).

Syntaxin-1A (STX1A) is a central component of the neuronal SNARE machinery. Together with SNAP-25 and VAMP2, STX1A supports docking and fusion of synaptic vesicles with the presynaptic membrane. Alterations in presynaptic proteins are expected in disorders characterized by axonal and terminal degeneration; therefore, reduced STX1A in PD may reflect synaptic loss rather than a primary pathogenic event. At the same time, the regulatory interactions of STX1A with exocytotic proteins and ion channels provide biologically plausible routes through which altered STX1A could modify neuronal vulnerability (Jahn et al., 2024; Südhof, 2014; Rizo, 2022).

This narrative review has three aims: first, to summarize the evidence for altered STX1A expression or function in PD; second, to evaluate proposed mechanisms while explicitly distinguishing direct evidence from extrapolation; and third, to assess the current limitations of STX1A as a biomarker or therapeutic target. In response to the limited disease-specific evidence, the review emphasizes uncertainty, causality, and experimental priorities rather than presenting a unified pathway as established fact.

## 2 Scope and literature approach

This article was prepared as a narrative review rather than a systematic review. Relevant studies were identified through focused searches of PubMed and reference lists using combinations of the terms “STX1A,” “syntaxin-1A,” “Parkinson disease,” “SNARE,” “synaptic vesicle,” “calcium channel,” “ferroptosis,” “neuroinflammation,” “extracellular vesicle,” and “biomarker.” Priority was given to original studies directly measuring STX1A in PD models or clinical samples, followed by mechanistic studies of STX1A in neuronal systems. Evidence from non-neuronal tissues was included only when clearly labeled as indirect or hypothesis-generating. Because the literature is heterogeneous and limited, no formal meta-analysis or risk-of-bias synthesis was undertaken.

## 3 STX1A biology relevant to PD

### 3.1 Molecular organization and conformational regulation

**FIGURE 1:**
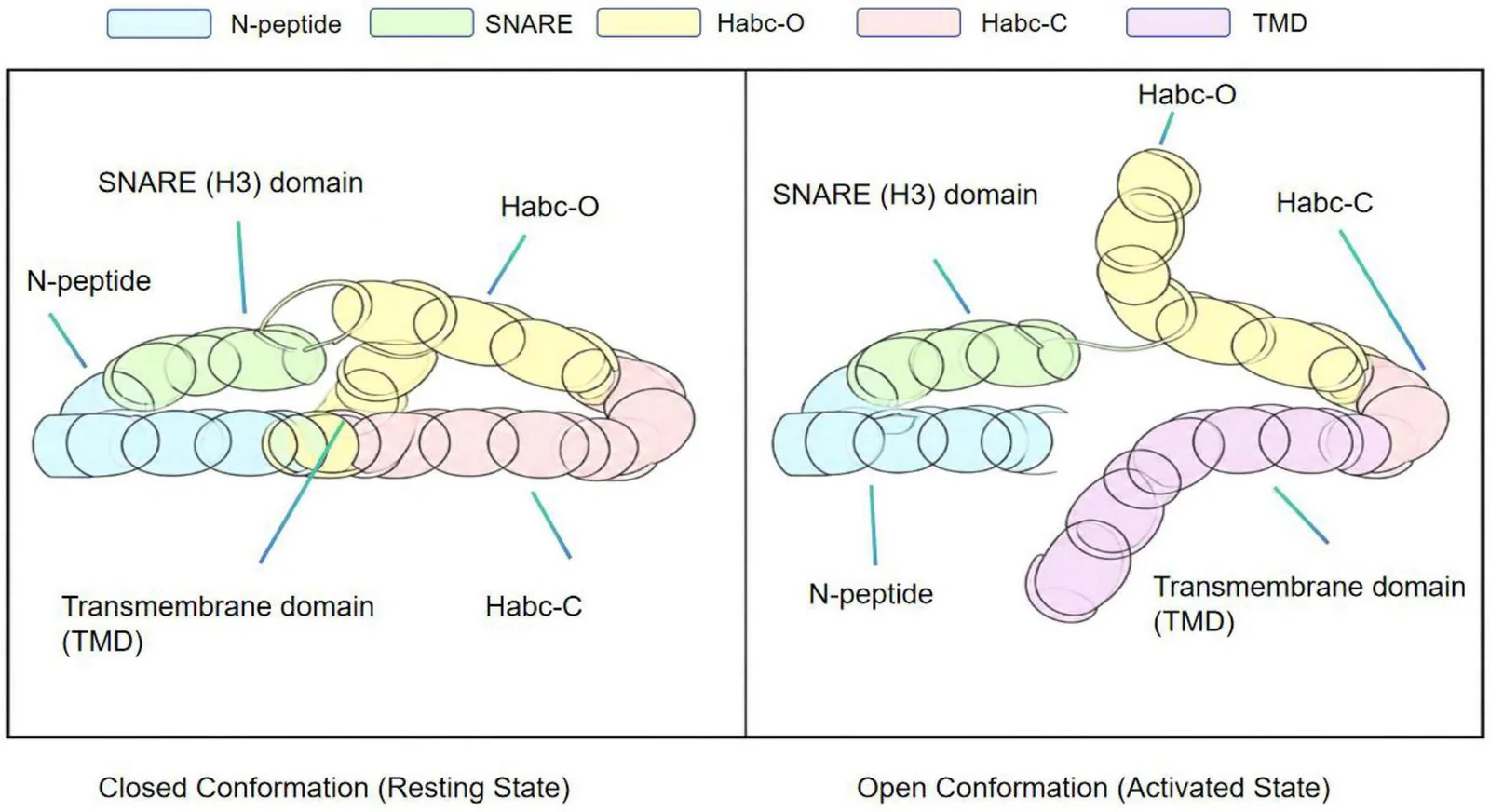
Conformational switch of STX1A. STX1A exists in two states: a closed conformation ***(left)*** where the Habc domain binds the SNARE domain to prevent premature assembly, and an open conformation (right) triggered by signals such as Ca^2+^, where the SNARE domain becomes available for SNARE complex formation and vesicle fusion.

Human STX1A is a 288-amino-acid membrane protein containing an N-peptide, an Habc regulatory domain, a SNARE motif (also termed the H3 domain), a polybasic juxtamembrane region, and a C-terminal transmembrane domain. Importantly, the two conserved transmembrane cysteines are established palmitoylation sites; they should not be described as the canonical VGCC-binding interface. Interactions with calcium channels are generally attributed to cytoplasmic regions of syntaxin and remain context dependent (Vardar et al., 2022) (Figure 1).

### 3.2 Synaptic vesicle fusion and neuronal function

Syntaxin-1A contributes one Qa-SNARE helix. Munc18-1, complexin, synaptotagmin-1, NSF, and α-SNAP coordinate assembly, calcium-triggered fusion, and recycling. Through these interactions, STX1A influences synaptic vesicle release. Genetic or functional disruption of STX1A can impair synaptic plasticity and neuronal development. Studies investigating STX1A-associated presynaptic regulation have further demonstrated its involvement in controlling neurotransmitter release and synaptic signaling processes (Marcelli et al., 2019). These established functions provide the biological background for examining STX1A in PD, but they do not by themselves demonstrate disease-specific causality (Ramakrishnan et al., 2020; Trimbuch and Rosenmund, 2016; Cheppali et al., 2025).

### 3.3 Ion-channel interactions: relevance and limitations

Syntaxin proteins interact with several voltage-gated calcium and potassium channels. The direction and magnitude of these effects vary with channel subtype, cellular context, and STX1A conformation. Evidence that Cav1.3-dependent pacemaking contributes to oxidative stress in substantia nigra neurons supports a general role for calcium burden in PD, but it does not directly show that STX1A downregulation disinhibits Cav1.3 in PD. Accordingly, the STX1A–VGCC relationship should be treated as a mechanistic question requiring direct electrophysiological validation (Trus and Atlas, 2024; Yeh et al., 2019).

## 4 Evidence linking STX1A to Parkinson’s disease

### 4.1 Animal and tissue-level evidence

Proteomic analysis of the substantia nigra in a unilateral 6-hydroxydopamine rat model reported reduced STX1A protein abundance. This observation is consistent with disruption of presynaptic machinery in a toxin-induced lesion. However, because 6-hydroxydopamine may contribute dopaminergic terminal and neuronal loss, reduced STX1A may reflect loss of presynaptic structures rather than an upstream molecular defect (Xiong et al., 2014).

### 4.2 Extracellular-vesicle evidence

Studies of neuron-derived extracellular vesicles have reported lower STX1A and higher oligomeric α-synuclein in PD. In a preliminary cohort of PD patients with probable REM sleep behavior disorder, STX1A concentrations were lower than in PD patients without probable REM sleep behavior disorder. These data suggest that extracellular-vesicle STX1A may reflect synaptic pathology or disease phenotype. The studies were relatively small, and their cross-sectional design does not determine whether STX1A changes precede neurodegeneration (Agliardi et al., 2021; Meloni et al., 2023).

### 4.3 Peripheral-blood evidence

A co-expression-network analysis of GEO datasets identified STX1A as a PD-associated module gene, followed by qRT-PCR and immunoblot validation of reduced STX1A in peripheral blood mononuclear cells. Immune-cell deconvolution also validated associations between STX1A-related modules and inferred peripheral immune-cell proportions. The sample size, demographic composition, treatment status, and validation strategy should be considered when interpreting these findings (Dong et al., 2023).

### 4.4 Summary of disease-specific evidence

Convergent evidence from animal models, extracellular vesicles, and peripheral blood studies has shown reduced STX1A in PD (Table 1). However, the causal direction and clinical significance of this association remain to be established.

**TABLE 1:** Summary of disease-specific evidence for STX1A in Parkinson’s disease (PD).

Study/context	Sample or model	Principal observation	Interpretive value	Major limitation
Xiong et al., 2014	6-OHDA rat substantia nigra	Reduced STX1A protein	Supports presynaptic disruption in a PD model	Toxin lesion and neuronal loss confound directionality
Agliardi et al., 2021	Serum neuron-derived extracellular vesicles	Lower STX1A; inverse relationship with oligomeric α-synuclein	Potential peripheral correlate of synaptic pathology	Small, cross-sectional cohort; limited specificity data
Meloni et al., 2023	PD with vs. without probable RBD	Lower extracellular-vesicle STX1A in probable RBD	Possible association with a more severe/prodromal phenotype	Questionnaire-defined subgroup and preliminary cohort
Dong et al., 2023	GEO datasets and PBMC validation	Reduced STX1A mRNA/protein; immune-module correlations	Hypothesis-generating peripheral evidence	No definitive diagnostic metrics or causal experiments
Hooshmand et al., 2022	PD patients and control subjects; peripheral blood/clinical cohort	Altered STX1A expression associated with PD-related molecular changes	Provides clinical evidence supporting STX1A dysregulation as a PD-associated molecular feature	Limited cohort size; peripheral findings may not fully reflect CNS pathology
Singer-Lahat et al., 2018	Human neuronal tissue and cellular synaptic models	STX1A abnormalities affect synaptic vesicle trafficking and neurotransmitter release	Supports STX1A as an essential regulator of presynaptic integrity and neuronal communication	Limited PD-specific validation and unclear causal relationship
Yang et al., 2015	PD-related experimental models and molecular studies	STX1A involvement in synaptic transmission and neuronal functional regulation	Provides mechanistic basis linking STX1A dysfunction with neuronal vulnerability	Mainly model-based evidence; human disease relevance requires confirmation

## 5 Mechanistic hypotheses and evidence gaps

### 5.1 Calcium dysregulation and mitochondrial stress

One proposed model Transfer the reference/figure/table citation(s) to the main text, as citations are not allowed in section headers.is that reduced STX1A alters the functional coupling between exocytosis and voltage-gated calcium channels, thereby increasing calcium burden in vulnerable dopaminergic neurons. This model is biologically plausible because substantia nigra neurons exhibit autonomous pacemaking. However, no study has yet demonstrated the sequence STX1A loss → VGCC disinhibition → calcium overload in a PD-relevant neuronal system. Direct testing requires STX1A knockdown or conditional deletion combined with channel-subtype-specific electrophysiology, calcium imaging, and rescue by re-expression of wild-type or interaction-deficient STX1A (Vardar et al., 2021; Chan et al., 2009; De Stefani et al., 2016).

### 5.2 Ferroptosis: a cross-tissue hypothesis

Ferroptosis has independent relevance to PD have been reported in experimental and clinical contexts. A recent gastric-cancer study linked STX1A depletion to mitochondrial dysfunction and ferroptosis. An STX1A–ferroptosis relationship in PD should be described as a testable cross-tissue extrapolation. Neuronal validation should measure lipid ROS, labile iron, GPX4 activity, mitochondrial respiration, and rescue by ferroptosis inhibitors after controlled STX1A manipulation (Niu et al., 2025; Dixon et al., 2012; Masaldan et al., 2019; Bai et al., 2021; Do Van et al., 2016; Flønes et al., 2024).

### 5.3 Neuroimmune regulation

Syntaxin-1A may influence neuroimmune signaling indirectly through altered neurotransmitter release and neuron–glia communication, suggest an association with systemic immune dysregulation. Nevertheless, correlation does not show that STX1A regulates immune-cell activation, antigen presentation, polarization, or cytokine secretion. Cell-resolved studies in microglia, monocytes, dendritic cells, and lymphocyte subsets are needed to determine whether STX1A has immune functions distinct from its neuronal role (Pajares et al., 2020; Dong et al., 2023).

### 5.4 Gut–brain axis as a future research direction

No direct evidence currently links STX1A to microbiota-driven pathology or gut–brain signaling in PD. Nevertheless, previous studies have demonstrated that gut microbiota can influence motor deficits and neuroinflammation in Parkinson’s disease models, highlighting the potential relevance of microbiota–brain interactions in PD pathophysiology (Sampson et al., 2016). Although STX1A is expressed in enteric neurons and microbial metabolites can influence neuronal and immune function, these observations do not establish an STX1A-dependent gut mechanism. The topic is therefore better positioned as a future direction: studies could examine STX1A expression in enteric neurons across PD stages and determine whether defined microbial metabolites alter STX1A abundance, localization, or SNARE function. Until such evidence is available, the gut–brain axis should not be presented as a co-equal established pathway (Braak et al., 2006; Loh et al., 2024; Barrenschee et al., 2017).

### 5.5 Cause, consequence, or biomarker?

**FIGURE 2:**
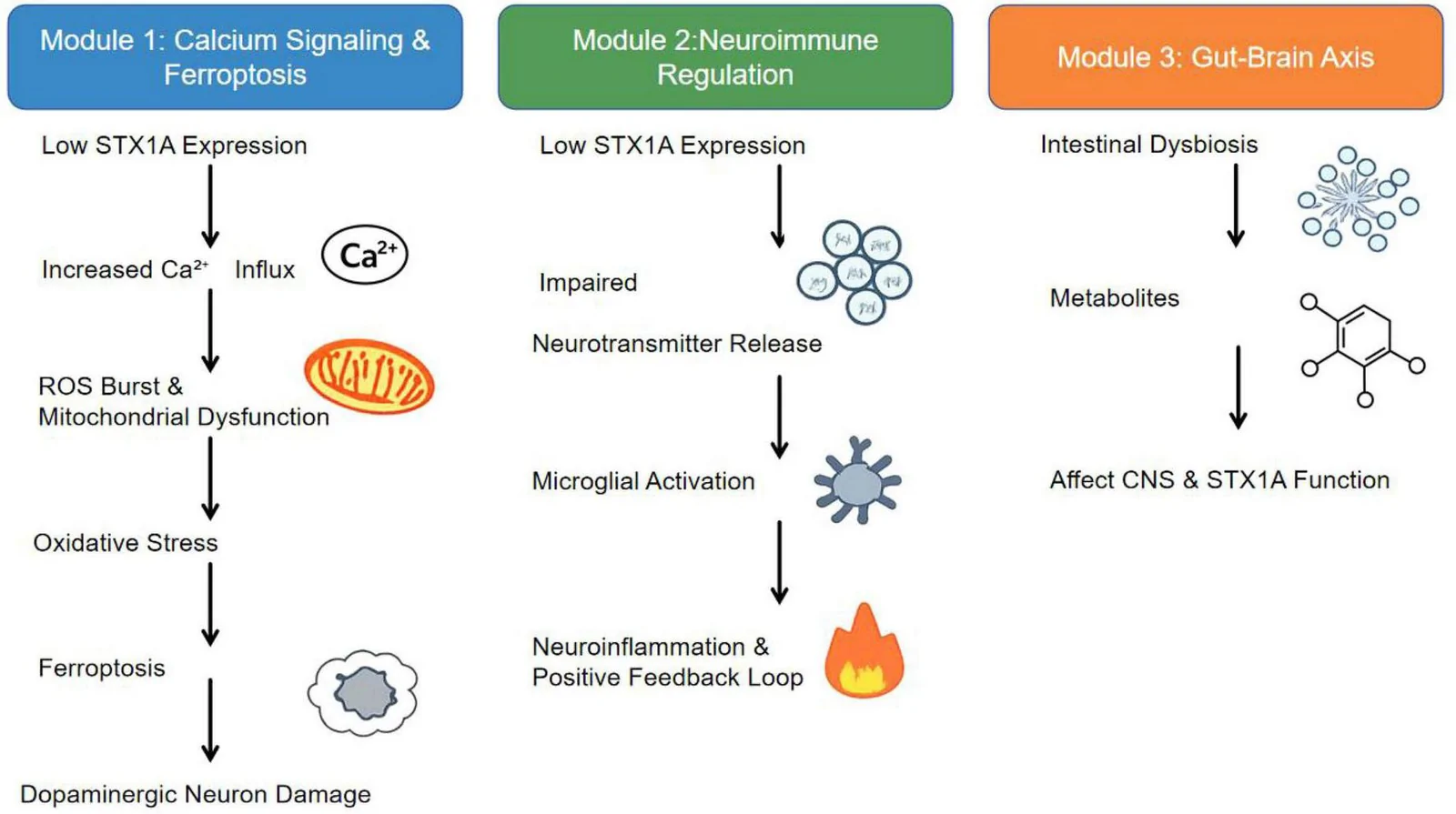
Evidence-graded model of STX1A in PD. STX1A downregulation is positioned as a central observation in PD. Arrows indicate proposed mechanistic links connecting reduced STX1A to calcium signaling dysregulation, ferroptosis, neuroimmune modulation, and gut-brain axis interactions. These pathways are presented as mechanistic hypotheses requiring direct experimental validation in PD-relevant models, rather than as established pathogenic cascades. The gut-brain axis is included as a future research direction, as direct evidence linking STX1A to microbiota-driven gut pathology in PD is currently lacking.

The central unresolved question is directionality. Reduced STX1A could be an upstream contributor that increases neuronal vulnerability, a downstream consequence of synaptic-terminal and dopaminergic-neuron loss, an epiphenomenon associated with altered cell composition, or a biomarker that tracks disease without participating in pathogenesis. To distinguish among these possibilities, future studies should employ cell-type-specific conditional knockout and overexpression models of STX1A to determine whether STX1A dysfunction directly affects neuronal vulnerability. Longitudinal sampling before the onset of substantial neuronal loss is necessary to establish the temporal sequence between STX1A alterations and neurodegeneration. In addition, rescue experiments should be performed to evaluate whether restoration of STX1A expression can reverse PD-relevant phenotypes. Electrophysiological studies are also required to determine whether STX1A deficiency alters synaptic transmission, calcium channel regulation, or neuronal excitability. Furthermore, mediation analyses integrating STX1A manipulation with key pathological processes, including calcium burden, synaptic release dysfunction, and dopaminergic neuronal survival, will be essential to clarify the molecular pathways through which STX1A may contribute to PD progression. Collectively, these approaches will help distinguish whether reduced STX1A represents a causal driver, a downstream consequence, or a disease-associated biomarker, thereby defining its precise biological role in PD (Figure 2).

## 6 Translational perspectives

### 6.1 STX1A as a candidate biomarker

Preliminary studies suggest that STX1A can be measured in peripheral blood mononuclear cells and neuron-derived extracellular vesicles. However, biomarker development requires substantially more evidence. Published studies have not established robust area-under-the-curve values, sensitivity, specificity, reproducibility across laboratories, or discrimination from multiple system atrophy, dementia with Lewy bodies, Alzheimer’s disease, inflammatory disorders, and other may contribute of neuronal injury. The relationship between circulating STX1A and brain pathology is also uncertain. STX1A should therefore be described as a candidate biomarker rather than a validated diagnostic or prognostic marker (Agliardi et al., 2021; Meloni et al., 2023; Dong et al., 2023).

Priority studies include assay standardization, blinded replication, multicenter cohorts, longitudinal sampling, adjustment for medication and disease duration, and comparison with established clinical and molecular biomarkers. Cell-composition effects in PBMCs and the cellular origin of extracellular-vesicle STX1A also need to be resolved.

### 6.2 Therapeutic implications

Strategies that restore STX1A expression or modify STX1A-related calcium signaling are currently hypothetical. Broad STX1A overexpression could disrupt normal neurotransmission because synaptic release depends on tightly regulated protein stoichiometry. Any gene-therapy approach would require cell-type-specific targeting, dose control, and demonstration that rescue occurs without seizures, excitotoxicity, or impaired synaptic plasticity.

Dihydropyridine calcium-channel blockers showed neuroprotective effects in preclinical models, but the phase 3 STEADY-PD III randomized trial found that immediate-release isradipine did not slow clinical progression in early PD. This negative result limits optimistic claims regarding VGCC-blocker repurposing and highlights the gap between mechanistic plausibility and clinical efficacy. Potential explanations–including insufficient brain exposure, dose limitations, channel-subtype selectivity, disease stage, and heterogeneity–remain under discussion but do not overturn the negative primary outcome (Parkinson Study Group STEADY-PD III Investigators, 2020).

Ferroptosis inhibitors are also experimental candidates. Their relevance to an STX1A-centered strategy depends first on demonstrating that STX1A manipulation changes ferroptotic susceptibility in dopaminergic neurons. Blood–brain barrier penetration, long-term safety, and effects on physiological iron and lipid metabolism would then require careful evaluation (Niu et al., 2025).

## 7 Conclusions and future directions

Syntaxin-1A is essential for presynaptic vesicle fusion, and several independent experimental or clinical studies have reported reduced STX1A in PD-related samples. These observations support an association between STX1A and PD-associated synaptic pathology. They do not yet demonstrate that reduced STX1A initiates or accelerates neurodegeneration. The strongest current interpretation is that STX1A is a candidate marker of altered presynaptic biology whose causal and clinical significance remains unresolved.

Mechanistic links to calcium dysregulation, mitochondrial dysfunction, ferroptosis, and neuroimmune signaling are plausible but unevenly supported. The gut–brain axis currently lacks direct STX1A-specific evidence. Future work should prioritize temporal and cell-type-specific causality, electrophysiological validation of channel interactions, neuronal testing of ferroptosis, functional studies in immune cells, and rigorous clinical biomarker validation. A clearer separation of observation, inference, and hypothesis will be essential for determining whether STX1A is merely reduced in PD or represents a biologically actionable component of disease.
